# Caring for Older Adults With Vision Impairment and Dementia

**DOI:** 10.1093/geroni/igaa043

**Published:** 2020-09-11

**Authors:** Varshini Varadaraj, Shang-En Chung, Kayla S Swiatek, Orla C Sheehan, Ashley Deemer, Joshua R Ehrlich, Jennifer L Wolff, Lama Assi, David L Roth, Bonnielin K Swenor

**Affiliations:** 1 Dana Center for Preventive Ophthalmology, Johns Hopkins Wilmer Eye Institute, Baltimore, Maryland; 2 Johns Hopkins University Center on Aging and Health, Baltimore, Maryland; 3 University of Kentucky College of Medicine, Lexington; 4 Division of Geriatric Medicine and Gerontology, Johns Hopkins University School of Medicine, Baltimore, Maryland; 5 Lions Vision Research and Rehabilitation Center, Wilmer Eye Institute, Johns Hopkins University School of Medicine, Baltimore, Maryland; 6 Department of Ophthalmology and Visual Sciences, Center for Eye Policy and Innovation, University of Michigan, Ann Arbor; 7 Institute for Health Care Policy and Innovation, University of Michigan, Ann Arbor; 8 Roger C. Lipitz Center for Integrated Health Care, and the Department of Health Policy and Management, Johns Hopkins Bloomberg School of Public Health, Baltimore, Maryland

**Keywords:** Caregivers stress, Caregiving—Informal, Vision loss

## Abstract

**Background and Objectives:**

Dementia and vision impairment (VI) are common among older adults but little is known about caregiving in this context.

**Research Design and Methods:**

We used data from the 2011 National Health and Aging Trends Study, a nationally representative survey of Medicare beneficiaries, linked to their family/unpaid helpers from the National Study of Caregiving. Vision impairment was defined as self-reported blindness or difficulty with distance/near vision. Probable dementia was based on survey report, interviews, and cognitive tests. Our outcomes included hours of care provided, and number of valued activities (scored 0–4) affected by caregiving, per month.

**Results:**

Among 1,776 caregivers, 898 (55.1%, weighted) assisted older adults without dementia or VI, 450 (21.9%) with dementia only, 224 (13.0%) with VI only, and 204 (10.0%) with dementia and VI. In fully adjusted negative binomial regression analyses, caregivers of individuals with dementia and VI spent 1.7 times as many hours (95% confidence interval [CI] = 1.4–2.2) providing care than caregivers of those without either impairment; however, caregivers of individuals with dementia only (95% CI = 1.1–1.6) and VI only (95% CI = 1.1–1.6) spent 1.3 times more hours. Additionally, caregivers of individuals with dementia and VI had 3.2 times as many valued activities affected (95% CI = 2.2–4.6), while caregivers of dementia only and VI only reported 1.9 times (95% CI = 1.4–2.6) and 1.3 times (95% CI = 0.9–1.8) more activities affected, respectively.

**Discussion and Implications:**

Our results suggest that caring for older adults with VI involves similar time demands as caring for older adults with dementia, but that participation impacts are greater when caring for older adults with both dementia and VI.

**Translational Significance:** When compared with caring for older adults with either dementia or vision impairment, caring for older adults with both dementia and vision impairment involves more hours of caregiving per month and further limits caregivers’ ability to participate in social activities. Low-vision rehabilitation and integration of low-vision services into the care of older adults with dementia and vision impairment may reduce caregiver burden.

## Background and Objectives

For many older adults, vision impairment and dementia are salient features of aging. As in most parts of the world, with the aging of the population in the United States (1), the number of older adults with these often co-occurring impairments is set to rise (2,3). With an increasing number of older adults, not only do concerns arise about appropriately meeting the care needs of individuals in late life, but also about the impact that providing care may have on caregivers. There has been considerable research describing the experience of caregivers of people with dementia, and it has been noted that these caregivers provide more help with daily activities and encounter more conflicts with their social activity than caregivers of people without dementia (4,5).

The caregiving experience of those caring for adults with vision impairment has also been explored, albeit to a lesser extent. This literature has largely focused on the impact of caregiving on psychosocial health. One study using a clinical sample found about one third of caregivers assisting a relative with vision impairment had symptomatology indicating a high risk of depression (6). Other studies conducted in visually impaired populations reported an increased risk of depression among caregivers who provided greater hours of care (7,8).

An overlooked aspect of the caregiving paradigm is the role of co-occurring dementia and vision impairment on the caregiving needs of older adults, and the impact on those caring for them. While multimorbidity or the coexistence of any two or more health conditions is a risk factor for disability in older adults, and therefore can be argued to be associated with a magnified caregiving need profile, co-occurring vision and cognitive impairments have been shown to have an especially strong impact on functioning (9,10). Although vision and cognitive impairments affect various areas of daily functioning such as self-care, mobility, and household activities, activity limitations are not always analogous. For example, mobility limitations are particularly relevant to adults with vision impairment while an impact on activities of daily living (ADLs) is more pertinent to cognitive impairment (9,10).

In addition, there is much emerging literature showing that vision impairment is a risk factor for cognitive decline and dementia (11,12). Despite evidence establishing the relationship between vision and cognition, they are often still considered as disparate elements when treating older adults. A better understanding of the interplay between vision and cognitive impairment and the impact of the resultant cumulative disability risk on caregiving needs is required. These data will provide knowledge to help plan for appropriate supportive strategies, respite care, and interventions to support caregivers while optimizing health in older people with these commonly coexisting multimorbidities.

To address this gap and build on our understanding of caregiving needs of older adults, we examined caregiving relationships for individuals with vision impairment and dementia using data from the National Health and Aging Trends Study (NHATS) and the National Study of Caregiving (NSOC). We hypothesized that family and unpaid caregivers caring for older adults with co-occurring dementia and self-reported vision impairment may experience greater time demands, and, relatedly, a more pronounced impact on participation in valued activities, beyond that predicted by caring for an individual with vision impairment or dementia alone.

## Research Design and Methods

### Study Population

This study used data from the 2011 round of the NSOC linked to the corresponding 2011 NHATS, which together provide cross-sectional care recipient and caregiver perspectives on late-life care at a national level. These data are de-identified and publicly available and therefore exempt from institutional review board approval.

#### National Health and Aging Trends Study

The NHATS (13) is a nationally representative survey of Medicare beneficiaries aged 65 years and older. Comprehensive information on health and function, performance in daily activities in the prior month, and assistance received are collected via in-person interviews with study participants (or proxy respondents). For NHATS participants who receive assistance, further details on the activities for which assistance is provided, the relationship to the person(s) providing assistance, and information on whether help is paid or unpaid are collected in a detailed helper roster (13).

#### National Study of Caregiving

The NSOC (14) is a nationally representative study of family and other unpaid caregivers to NHATS participants which is conducted in conjunction with NHATS. National Study of Caregiving participants are identified from the NHATS helper roster on the basis of providing assistance with mobility, self-care, household activities, transportation, or medically oriented tasks to NHATS participants. Caregivers of community-dwelling NHATS participants receiving help with (a) self-care, (b) mobility, or (c) household (latter for health or functioning-related reasons specifically) activities, or those who lived in a residential care facility with supportive services, are eligible for the NSOC. National Study of Caregiving data include information from telephone interviews conducted with up to five helpers (five selected at random if more than five) to these NHATS participants.

#### Analytic sample

The 2,423 NHATS participants who were eligible (13,14) for NSOC had 4,935 caregivers who met eligibility criteria in 2011. National Study of Caregiving interviews were conducted with a total of 2,007 caregivers of 1,369 older adults (15). National Study of Caregiving respondents providing care to community-dwelling older adults were included (i.e., older adults in residential care facilities reliant on the availability of supportive services were excluded) leaving a sample of 1,786 family and unpaid caregivers (1,684 = spouse or son/daughter and 102 = unpaid relative) to 1,199 NHATS participants. Finally, we limited this study to all caregivers who had assisted an NHATS participant with any activity in the last month to yield an analytic sample of 1,776 caregivers linked to 1,196 NHATS participants.

### Caregiving Outcomes

Two caregiving outcomes were analyzed: the number of hours of care provided in the last month and the number of valued activities affected by caregiving (scored 0–4). Participation restriction in valued activities refers to activities reported as being very or somewhat important to the caregiver which were limited in the prior month because of caregiving. Valued activities included (a) visiting friends and family, (b) going out for enjoyment, (c) attending religious services, and (d) participating in club meetings or group activities. Participation restriction in each valued activity was coded as a binary variable (yes/no) and then summed to obtain the 0–4 composite score for this outcome.

### Vision

In NHATS, older adults provided self-reports of visual function for distance and near tasks, while using contact lenses or glasses (if necessary). For distance vision, participants were asked if they could see well enough to “recognize someone across the street” or “watch television across the room.” For near vision, participants were asked if they could “see well enough to read newspaper print.” As in prior studies (16,17), self-reported vision impairment was defined as participant- or proxy-reported blindness or difficulty with distance or near vision (i.e., answering “no” to either question).

### Dementia

Participants were classified as having probable, possible, or no dementia based on previously defined criteria (18,19) including survey report, the eight-item Informant Interview to Differentiate Aging and Dementia (AD8) criteria, and cognitive performance tests. National Health and Aging Trends Study respondents were classified as having probable dementia based on (a) participant- or proxy-reported physician diagnosis of dementia or Alzheimer’s disease, or (b) an AD8 score ≥2; the AD8 is an eight-item questionnaire administered to informants to assess memory, temporal orientation, judgment, and function of the participant (20), or (c) participant cognitive test scores ≤1.5 *SD*s below mean in at least two of the three cognitive domains—memory (immediate and delayed 10-word recall), orientation (date, month, year, and day of the week; naming the President and Vice President), and executive function (clock drawing test). Possible dementia was indicated by impairment (cognitive test scores ≤1.5 *SD*s below mean) in one domain in absence of meeting the physician diagnosis or AD8 criteria described above. Participants not meeting these criteria were classified as having no dementia.

In primary analyses, we considered only those with probable dementia (comparison group: possible dementia and no dementia), a narrower and more specific definition (19). In a sensitivity analysis, we combined probable and possible dementia to indicate any dementia (comparison group: no dementia), a broader and more sensitive definition.

### Other Covariates

Sociodemographic and health characteristics of older adults were drawn from NHATS (age, race/ethnicity, sex, marital status, income, comorbidities, and diabetes) and of caregivers were drawn from NSOC (age, sex, education, self-reported health status, relationship to the older adult, cohabiting status/travel time to older adults’ residence, duration of caregiving, activities for which assistance was provided including help with instrumental ADLs, health system interactions, and specific health management tasks). Older adults’ comorbidities included hypertension, arthritis, osteoporosis, lung disease, stroke, heart disease, cancer, depression, and fracture and were categorized as 0–1, 2–3, and ≥4 conditions. Diabetes (survey-reported physician diagnosis) was adjusted for separately as it is expected to be a strong confounder of the vision–caregiving relationship; diabetes is a driver of vision impairment (21) and diabetics may require more caregiving (22).

### Statistical Analysis

Sociodemographic and health characteristics were summarized across the four NHATS participant groups with and without vision impairment and dementia. Unweighted frequencies and weighted percentages for categorical variables and means (standard error [*SE*]) for continuous variables are reported. Multinomial logistic regression models were constructed to assess the differences in sociodemographic and health characteristics across the four NHATS participant groups (participants with and without vision impairment and dementia). Similarly, NSOC caregiver characteristics were summarized across these four groups (those caring for participants with and without vision impairment and dementia).

The nature and intensity of care provided by each group as expressed by hours of care per month, types of activities for which help is provided, health system interactions, and health management tasks are described. Caregiving-related participation effects in valued activities and the use of supportive services, stratified by the four NHATS participant groups with and without vision impairment and dementia, are also described.

Weighted (using NSOC weights) negative binomial regression models, clustered by NHATS participants (to account for correlation between multiple caregivers for an NHATS older adult), were constructed to examine how vision impairment and dementia status are associated with the intensity of caregiving (hours of caregiving in the last month) and the impact on participation in valued activities (number of valued activities affected by caregiving in the last month). This approach was chosen as caregiving hours and the number of valued activities affected by caregiving are both count data, and the expected value was not equal to the variance. Finally, models were constructed to test for interactions between impairment categories (i.e., vision impairment × dementia) to examine if co-occurring vision impairment and dementia affect caregiving outcomes in a nonadditive manner. All models were adjusted for NHATS participant age, race/ethnicity, sex, marital status, income, comorbidities, diabetes, and NSOC caregiver age, caregiver sex, caregiver education, caregiver’s self-reported health, caregiver relationship to the older adult, and cohabiting status. Covariates were included based on clinical relevance and/or previous demonstration of impact on vision impairment or dementia and caregiving needs.

Analyses were conducted in SAS 9.4 (SAS Institute, Cary, NC) and STATA 15 (StataCorp LLC, College Station, TX).

## Results

### Caregiver Characteristics

Among caregivers to older adults in 2011, 55.1% (95% confidence interval [CI] = 50.1–60.2) assisted older adults without dementia or vision impairment, 21.9% (95% CI = 18.1–25.6) with dementia only, 13.0% (95% CI = 10.7–15.3) with vision impairment only, and 10.0% (95% CI = 7.2–12.9) with dementia and vision impairment (Table 1). The majority (68.5%) of older adults had a single caregiver included from the NSOC sample, while 31.5% had more than one caregiver.

**Table 1.** Characteristics of NSOC Caregivers Linked to NHATS and Caregiving Activities

Caregiver characteristics	Total (*n* = 1,776)	No vision impairment or dementia (*n* = 898, 55.1%)	Dementia only (*n* = 450, 21.9%)	Vision impairment only (*n* = 224, 13.0%)	Vision impairment and dementia (*n* = 204, 10.0%)	*p* Value
Age, mean (*SE*)	57.0 (0.7)	57.8 (0.7)	57.4 (1.1)	54.2 (1.6)	55.4 (1.2)	.117
Female, %	62.3	60.4	66.3	61.1	65.8	.230
Education, %						.596
≤High school	42.9	419	41.0	45.5	49.3	
Some college	33.1	32.1	37.2	32.6	30.6	
≥College	24.0	26.0	21.8	21.9	20.1	
Self-rated health status, %						.254
Excellent or very good	50.5	53.3	46.9	47.8	46.5	
Good	28.5	26.5	31.9	32.7	27.5	
Fair or poor	21.0	20.3	21.2	19.5	26.1	
Relationship to the older adult, %						<.001
Spouse	23.0	28.2	18.6	15.3	13.6	
Daughter or son	53.0	48.3	60.8	56.0	58.0	
Other relative	16.4	14.6	15.5	21.2	22.0	
Nonrelative	7.7	9.0	5.1	7.5	6.4	
Travel time to older adults’ residence, %						.072
Co-reside	51.7	50.9	50.9	55.1	53.3	
≤10 min	25.5	26.8	26.0	24.1	18.6	
11–30 min	16.2	15.9	15.2	13.1	24.2	
≥31 min	6.7	6.5	7.9	7.7	3.9	
Duration of caregiving in years, %						.031
<1	16.6	18.5	16.2	12.7	11.7	
1–4	39.6	37.7	46.3	38.7	36.3	
>4	43.9	43.8	37.5	48.6	51.9	
Providing assistance for, %						
Shopping	90.8	91.3	89.2	91.4	91.1	.822
Transportation	87.3	88.4	85.5	90.0	81.7	.201
Housework	84.9	83.6	89.5	83.7	83.5	.303
Mobility	72.0	67.4	73.4	78.7	85.1	<.001
Banking	58.4	54.4	65.9	62.1	59.0	.047
Self-care	51.4	43.6	60.5	50.4	75.7	<.001
Health system logistics, %						
Make appointments	59.6	52.2	75.1	59.0	67.0	<.001
Order medicines	52.0	45.1	63.8	52.4	64.1	<.001
Handle insurance issue	39.9	36.5	43.9	40.0	50.0	.048
Health management, %						
Diet	30.6	30.4	31.1	30.0	31.4	.994
Foot care	29.7	25.8	29.9	34.4	44.2	<.001
Skin care	25.6	22.8	25.2	26.0	41.1	<.001
Exercise	23.1	20.1	28.5	19.3	32.6	.012
Dental care	15.7	9.7	23.1	11.8	37.9	<.001

*Note:* NHATS = National Health and Aging Trends Study; NSOC = National Study of Caregiving.

When compared with NHATS participants receiving care without dementia or vision impairment, those with dementia and vision impairment were older, less likely to be white or married, and more likely to have diabetes and a lower income (Supplementary Table 1). When compared with caregivers of older adults without either impairment, caregivers of participants with dementia and vision impairment were less likely to be a spouse, more likely to be an adult child, and more likely to have been providing care for longer than 4 years (Table 1).

### Caregiving Circumstances and Activities

Caregivers of persons with dementia and vision impairment were more likely to assist with mobility, banking, and self-care-related activities, as well as with activities pertaining to navigating health system logistics and health management than caregivers of persons without either impairment (Table 1).

### Caregiving-Related Difficulties

Caregivers of older adults with dementia and vision impairment were more likely to report reduced participation in each of the following activities: visiting friends and family, going out for enjoyment, attending religious services, and participating in club meetings or group activities (Table 2). Caregivers of persons with dementia and vision impairment were more likely to use one or more supportive services than caregivers of persons without either impairment.

**Table 2.** Caregiving-Related Difficulties Among Caregivers

Caregiver characteristics	Total (*n* = 1,776)	No vision impairment or dementia (*n* = 898, 55.1%)	Dementia only (*n* = 450, 21.9%)	Vision impairment only (*n* = 224, 13.0%)	Vision impairment and dementia (*n* = 204, 10.0%)	*p* Value
Impact on valued activities^a^, %						
Visiting friends and family	17.6	12.6	21.3	17.9	36.5	<.001
Going out for enjoyment	12.1	7.3	15.5	11.4	31.3	<.001
Attending religious services	8.1	5.4	11.6	8.6	14.5	<.001
Participating in club meetings or group activities	7.5	5.2	11.8	4.5	15.3	<.001
Works for pay, %	39.5	37.4	41.8	43.5	41.3	.436
Impact on work (among those who work), %						
Missed work in past month^b^	10.2	9.4	12.3	9.8	10.2	.859
Missed hours^c^	10.5	10.0	13.5	7.7	10.2	.647
Reduced productivity	14.0	11.9	18.7	12.8	15.5	.348
Supportive services or assistance directed to caregiver, %						
Respite care	10.6	7.2	16.3	9.6	18.6	<.001
Received training on how to assist	6.8	4.9	9.4	6.4	12.0	.004
Support group participation	4.6	3.9	7.9	2.3	4.0	.306
Use of ≥1 supportive service	18.4	13.1	27.8	16.5	29.5	<.001

^a^Reduced participation in the past month because of caregiving for activities identified as being somewhat or very important.

^b^Missed work refers to any missed time from work in the past month because of caregiving.

^c^Missed hours reflect hours of work missed because of caregiving in relation to all hours typically worked.

### Caregiving Intensity and Impact on Valued Activities

In unadjusted analyses examining group differences, caregivers assisting older adults without either impairment provided a mean of 64.9 (*SE* = 3.9) hours in the past month, 91.5 (*SE* = 5.8) hours for those with dementia only, 100.3 (*SE* = 13.7) hours for those with vision impairment only, and 125.1 (*SE* = 16.1) hours for those with co-occurring impairments. In regard to the impact on valued activities, caregivers assisting older adults without dementia or vision impairment, and with dementia only, vision impairment only, and co-occurring impairments, reported that participation in a mean of 0.30 (*SE* = 0.03), 0.59 (*SE* = 0.06), 0.42 (*SE* = 0.05), 0.97 (*SE* = 0.10) valued activities were affected in the last month due to providing care, respectively.

In weighted, fully adjusted negative binomial models, in the last month, caregivers of older adults with dementia and vision impairment spent 1.7 times more hours on caregiving (incident rate ratio = 1.7; 95% CI = 1.4–2.2), and caregivers of adults with dementia only (95% CI = 1.1–1.6) and vision impairment only (95% CI = 1.05–1.61) spent 1.3 times more hours, when compared with caregivers of older adults without either impairment (Table 3). Additionally, caregivers of older adults with dementia and vision impairment reported 3.2 times as many valued activities were affected per month (95% CI = 2.2–4.6) when compared with caregivers of older adults without dementia and vision impairment, while caregivers of those with dementia only reported 1.9 times (95% CI = 1.4–2.6) more activities per month were affected. Although not statistically significant, the caregivers of older adults with vision impairment had 1.3 times (95% CI = 0.9–1.8) more activities affected per month.

**Table 3.** Regression Analysis: Caregiving Outcomes for Caregivers of Older Adults by Vision Impairment and Probable Dementia, Accounting for Clustering by Care Recipient

*N* = 1,776	Model 1. Caregiving hours per month	Model 2. Number of valued activities affected due to providing care
*Group*	*IRR (95% CI)*	*IRR (95% CI)*
No vision impairment or dementia	Reference	Reference
Dementia only	**1.3 (1.1–1.6)**	**1.9 (1.4–2.6)**
Vision impairment only	**1.3 (1.1–1.6)**	1.3 (0.9–1.8)
Vision impairment and dementia	**1.7 (1.4–2.2)**	**3.2 (2.2–4.6)**

*Notes*: Bold values indicate *p* < .05. CI = confidence interval; IRR = incident rate ratio; NHATS = National Health and Aging Trends Study; NSOC = National Study of Caregiving. Models adjusted for NHATS participant age, race/ethnicity, sex, marital status, income, comorbidities, and diabetes, and NSOC caregiver age, caregiver sex, caregiver education, caregiver self-reported health, caregiver relationship to the older adult, and cohabiting status.

In other models testing for interactions between each impairment category, the interaction terms were not statistically significant (*p* > .05 for both models of caregiving outcomes; Supplementary Table 2). In additional sensitivity analysis reclassifying dementia to include probable and possible dementia, the results were similar.

## Discussion and Implications

In a nationally representative sample of family and unpaid caregivers who assist older adults with mobility, self-care, household activities, transportation, and medical care, we found that caregivers of individuals with dementia and self-reported vision impairment spent almost twice as many hours providing care than caregivers of older adults without dementia or self-reported vision impairment, and they had three times as many valued activities affected. Caregivers of individuals with either dementia or self-reported vision impairment still spent 1.3 times more hours and had about 1.9 to 1.3 times as many valued activities affected when compared with caregivers of older adults without these impairments. These results indicate that caring for older adults with self-reported vision impairment places similar demands on family and relatives as does caring for older adults with dementia and suggest that these implications are additive when caring for older adults with both dementia and vision impairment.

Vision and cognitive impairments share common risk factors (23,24) and vision impairment itself is a risk factor for cognitive decline (11). Prior research has shown that older adults with dementia have a higher prevalence of age-related vision problems than the general population (25). Alzheimer’s disease, the most common cause of dementia in older adults, progressively impairs cognition. Associated memory loss, disorientation, emotional disturbances, and impairment of judgment (26) may necessitate assistance from family and other caregivers to perform daily tasks. In combination with the diminution in visual function, which commonly accompanies Alzheimer’s and includes contrast sensitivity loss (27), visual field defects (28), delayed eye saccades (29), and impaired object recognition (30), difficulties faced with ADLs may be exacerbated (9,31). As a corollary, our results confirm that older adults with co-occurring vision impairment and dementia require a greater intensity of caregiving, and caring for them places more demands on family caregivers.

The caregiving effects of dementia have been well documented. Collectively, this research shows that caring for persons with dementia takes up more time, requires help across a wider range of daily activities, and has a greater impact on family caregivers’ social life, physical health, and mental well-being (15,32–34). However, our results show that caring for older adults with self-reported vision impairment imposes consequential caregiving demands.

The few studies examining caregiving needs among older adults with vision loss have been largely composed of small clinic-based cohorts. Bambara et al. (6) found that among 96 caregivers of patients attending a low-vision rehabilitation clinic, 35% were identified at risk for depression. Caregivers at risk for depression were younger, more likely to be female, caring for a relatively younger person with vision loss, and providing assistance for a loved one with worse visual acuity compared with nondepressed caregivers. Braich et al. (7) conducted a study on 522 individuals in India providing care to legally blind (visual acuity varied from 20/200 to no light perception) relatives. Using the Burden Index of Caregivers, a questionnaire that measures the time, emotional, physical, and existential demands of caregiving, they ascertained that more severe forms of blindness were associated with requiring more help with ADLs and additional hours of close supervision per day, both of which increased risk of caregiver depression. Another clinic-based study in Canada of 236 caregivers found worse Burden Index of Caregivers scores among those caring for persons with poorer vision (20/200 or worse) than compared to those caring for persons with better vision (visual acuity 20/60 to 20/200) (8). Taking together these two streams of caregiving data on cognitive and vision impairments, it follows that individuals caring for older adults with co-occurring dementia and vision impairment are most likely to provide more intense care and experience a greater restriction of their social activities. Care activities for older adults with vision impairment are likely different from those of older adults with dementia, and therefore, demands may be “additive” for adults with both types of impairments. For example, older adults with vision impairment may require greater assistance with mobility-related activities while dementia may necessitate more help with ADLs and self-care.

This study has some limitations. First, our estimates of vision impairment from NHATS participants/proxies and data on caregiving hours and impacted activities from NSOC caregivers were based on self-reported data that may be subject to recall bias. However, self-reported vision still provides valuable information that captures individuals’ perspectives on their disability and function and has been previously used in NHATS (16,17). To define dementia, the use of multiple criteria that have been previously employed (35), including self-report of diagnosis, use of the AD8 instrument, and cognitive performance tests, is a strength of the study. Regardless, this definition is subject to measurement error, because we relied on interviewer-reported diagnosis and a nonclinical assessment. Finally, causal associations cannot be determined from this cross-sectional study, and longitudinal research is needed to determine causal processes underlying the observed effects. Relatedly, because vision impairment and dementia are both progressive in nature, these observed associations will likely change over time.

Despite these limitations, this study is among the first to provide insight into the caregiving needs and relationships of older adults at the crossroads of vision impairment and dementia. Our results show that caring for older adults with vision loss places similar time demands on caregivers when compared with caring for older adults with dementia. However, caring for older adults with co-occurring dementia and vision loss is associated with enhanced caregiving demands as evidenced by greater intensity of providing care as well a greater restriction of participation in caregiver valued personal/social activities. Given that older adults with these commonly co-occurring impairments have more complex caregiving needs and require greater intensity and breadth of assistance, their family and unpaid caregivers need to be better engaged and supported by stakeholders in the health care system. Outreach programs that focus on caregiving training and support services could help educate caregivers on caring for these older adults at increased risk of functional decline as well as provide respite services to reduce the strain experienced by them. In addition, better utilization of low-vision rehabilitation and integration of low-vision services into the overall care of older adults may be needed. Future research should also examine how caregiver support may be useful in improving caregiver quality of life and the quality of care provided.

## Supplementary Material

igaa043_suppl_Supplementary_Tables
