# Supplementary material for: Caring for Older Adults With Vision Impairment and Dementia
Source: Innov Aging. 2020 Sep 11;4(6):igaa043. doi: 10.1093/geroni/igaa043 (PMC7657090; doi:10.1093/geroni/igaa043)
Supplement: igaa043_suppl_Supplementary_Tables [file igaa043_suppl_supplementary_tables.docx]

Supplementary Table 1. Characteristics of NHATS Participants Linked to NSOC Caregivers.

| Participant Characteristics | Total  (n=1196) | No vision impairment or dementia  (n=617, 57.8%) | Dementia only  (n=298, 20.9%) | Vision impairment only  (n=143, 11.9%) | Vision impairment and dementia  (n=138, 9.3%) | p-value |
| --- | --- | --- | --- | --- | --- | --- |
| Age, mean (SE) | 78.4 (0.2) | 76.5 (0.4) | 81.9 (0.4) | 78.2 (0.5) | 82.5 (0.5) | <.001 |
| Female, % | 65.0 | 65.9 | 65.7 | 63.3 | 60.0 | 0.571 |
| Race/Ethnicity, %  Non-Hispanic white  Non-Hispanic black  Hispanic/Other | 74.9  13.2  12.0 | 78.3  11.7  10.0 | 67.9  15.4  16.8 | 75.5  14.7  9.8 | 68.7  15.5  15.8 | 0.029 |
| Income  <$15,000  $15,000-$29,999  $30,000-$59,999  ≥$60,000 | 35.0  29.5  21.2  14.3 | 29.4  28.5  23.2  18.9 | 42.3  27.0  22.2  8.5 | 37.6  36.3  18.0  8.2 | 49.8  33.0  10.5  6.7 | <.001 |
| Marital Status, %  Married/living with partner  Separated/divorced  Widowed/never married | 51.5  9.1  39.3 | 58.0  9.1  33.0 | 43.8  10.6  45.7 | 42.6  10.3  47.1 | 40.6  4.6  54.8 | <.001 |
| No. of comorbidities, %^a^  0-1  2-3  4+ | 12.1  47.2  40.8 | 12.8  48.9  38.3 | 13.2  45.0  41.8 | 9.9  42.5  47.6 | 7.5  47.3  45.2 | 0.209 |
| Diabetes, % | 36.3 | 39.0 | 27.1 | 42.6 | 32.2 | 0.009 |

**^a^** Comorbid conditions include hypertension, arthritis, osteoporosis, lung disease, stroke, heart disease, cancer, depression, and hip fracture.

Supplementary Table 2. Regression Analysis: Testing for Interactions Between Vision Impairment and Probable Dementia for Caregiving Outcomes.

| N=1,776 | **Model 1. Caregiving hours per month** | **Model 2. Number of valued activities affected due to providing care** |
| --- | --- | --- |
|  | *IRR (95% CI)* | *IRR (95% CI)* |
| Vision impairment only | 1.3 (1.1, 1.6) | 1.3 (0.9, 1.8) |
| Dementia only | **1.3 (1.1, 1.6)** | **1.9 (1.4, 2.6)** |
| Vision impairment X Dementia  (Interaction term) | 0.9 (0.7, 1.4) | 1.3 (0.8, 2.2) |

Note: IRR=incident rate ratio; CI=confidence intervals.

Models adjusted for NHATS participant age, race/ethnicity, sex, marital status, income, comorbidities, and diabetes, and NSOC caregiver age, caregiver sex, caregiver education, caregiver self-reported health, caregiver relationship to the older adult, and cohabiting status.
